# Atomistic study of the solid state inside graphene nanobubbles

**DOI:** 10.1038/s41598-017-18226-9

**Published:** 2017-12-20

**Authors:** Evgeny Iakovlev, Petr Zhilyaev, Iskander Akhatov

**Affiliations:** Center for Design, Manufacturing and Materials, Skolkovo Institute of Science and Technology, Moscow, Russia

## Abstract

A two-dimensional (2D) material placed on an atomically flat substrate can lead to the formation of surface nanobubbles trapping different types of substances. In this paper graphene nanobubbles of the radius of 7–34 nm with argon atoms inside are studied using molecular dynamics (MD). All modeled graphene nanobubbles except for the smallest ones exhibit an universal shape, i.e., a constant ratio of a bubble height to its footprint radius, which is in an agreement with experimental studies and their interpretation using the elastic theory of membranes. MD simulations reveal that argon does exist in a solid close-packed phase, although the internal pressure in the nanobubble is not sufficiently high for the ordinary crystallization that would occur in a bulk system. The smallest graphene bubbles with a radius of 7 nm exhibit an unusual “pancake” shape. Previously, nanobubbles with a similar pancake shape were experimentally observed in completely different systems at the interface between water and a hydrophobic surface.

## Introduction

Graphene is a basic material used to create various heterostructures that have been actively studied and have shown potential for many electronic and optical applications^1–4^. These structures are usually created layer by layer, and sometimes, atoms and molecules can be trapped between layers, which results in bubbles, blisters and nanodrums of nanoscale height^5–10^. The formation of these enclosures were first considered undesirable defects of the surface. However, later studies showed that they possess several interesting properties, including a giant pseudo magnetic field^5,6^ and extreme internal pressure^7–9^, and their presence can adjust phonon-electron interactions^11^. These structures can also be used as hydrothermal reactors^12^, as surface modifiers^7^ and for the visualization of chemical reactions^13^. Graphene nanobubbles and blisters on metalic substrates produced by bombarding with noble gas ions^14,15^ are actively studied and could be used for novel gas storage technologies.

A high pressure of up to 1 GPa^9^ is assumed to be present in the substance trapped inside the heterostructures. These pressures can lead to a phase transition that is strongly affected by the surface interaction between the substrate and the two-dimensional (2D) crystal. This confinement ordering has already been discovered in carbon nanotubes both experimentally^16^ and theoretically^17^. However, because of the small radius of the nanotube and, consequently, the small number of atoms and molecules inside it, it is more appropriate to treat such a phase transition as an ordering of atoms and molecules. The phase transition inside nanobubbles leads to intermediate states wherein the system consists of billions of atoms, and its thermodynamic behavior is understood and largely determined by the interaction with the interface.

The pressure inside graphene nanobubbles is determined by their shape and hence could be experimentally evaluated using atomic force microscopy (AFM). In the work^8^ the consistent experimental AFM study of geometry of nanobubbles in 2D materials was performed. It was shown that the shape of the nanobubbles with the radius more than 50 nm exhibits universal scaling, i.e. the ratio of the bubble height (H) to the radius of its footprint (R) is constant in a wide range of bubble sizes and depends only on type of 2D material used (BN, MoS_2_ or graphene). In addition to these experiments, the elastic theory of membranes was used for theoretical calculation of nanobubble shape and mechanical properties. This theory validates the experimental observations and yields the expression for the ratio of the maximum nanobubble’s height to the radius of it’s footprint (H/R) for round-type nanobubbles. In the experiments^8^ the substance inside the nanobubbles was unknown. Therefore there was no possibility to estimate adhesion energy magnitude between trapped substance and 2D-crystal or substrate which was used as a parameter in the elastic theory of the membranes. Instead, the adhesion energy was fitted so that theoretically predicted H/R ratio matches with experimental measurements.

Another issue associated with application of the elastic theory of membranes to modeling of graphene nanobubbles is related to the fact that all material properties, such as Young’s modulus and adhesion energies, are assumed to be constant. It is not obvious. In fact, one study^18^ showed that to correctly apply this theory to atomically thin and micron-sized membranes, the dependence of Young’s modulus and bending rigidity on the size of the system should be accounted for. Therefore, verifying the elastic theory of membranes with the help of molecular dynamics (MD) will be useful. In atomistic modeling, all material parameters can be calculated, and the graphene nanobubble takes shape spontaneously. In general, MD simulations could play a valuable role in evaluating the assumptions of the elasticity theory of nanoscale membranes and in providing a benchmark system for validating analytic theories.

In this paper, the MD method is used to investigate graphene nanobubbles. Here we use a graphene sheet with graphite substrate. Together with the substance trapped inside, these two parts compose the graphene nanobubble. Nanobubbles with radii in the range of 7–34 nm are studied. The first goal of this paper is to study state of the matter inside graphene nanobubbles, and in particular, to show if solid state of the matter can be observed. The second goal is to study the nanobubbles shape and to see if it fits to the predictions of the standard theory of elastic membranes^8^.

It is shown that argon is in the solid state of the matter with a close-packed crystal structure in all the nanobubbles with various radii. The thermodynamic states of the observed crystal structures in the pressure-temperature (P-T) diagram are located considerably below the melting curve of the bulk argon. We attribute this behavior to the stabilizing role of the interface between the 2D crystal and the substrate. It is also shown that the results provided by theory of elastic membranes agree well with those obtained by atomistic modeling. We also report here the existence of so-called “pancake” nanobubbles, which have not been observed in heterostructures yet, and which contradicts the theory of elastic membranes, but have been found at the interface between highly oriented pyrolytic graphite and water^19^.

## Methods

**Figure 1:**
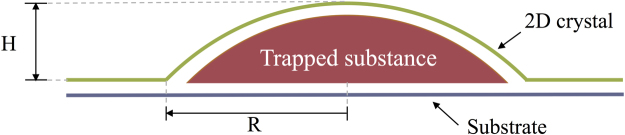
Schematic representation of a meridional 2D slice of the graphene nanobubble. Trapped substance: argon atoms; 2D crystal: curved graphene sheet; Substrate: graphene sheet frozen in the direction perpendicular to the substrate; H: maximum height, and R: radius of the nanobubble, both measured for the undisturbed 2D crystal.

The MD method is used to study the properties of graphene nanobubbles. The model of the graphene nanobubble is presented schematically in Fig. 1. The model consists of three parts: a 2D crystal, i.e., the graphene sheet, a trapped substance, i.e., the argon atoms, and a substrate, i.e., another graphene sheet.

The carbon-carbon interaction is described by the adaptive intermolecular reactive bond order potential (AIREBO)^20^ which is known to describe well the mechanical properties of graphene and graphite^21^. The interaction between argon atoms is described by Lennard-Jones potential (see Table 1). The interaction between carbon-argon atoms is also described by the Lennard-Jones potential, the parameters of which are adjusted using Lorentz-Berthelot combining rules^22^:

1 $$\begin{array}{c}{\sigma }_{ij}=\frac{{\sigma }_{i}+{\sigma }_{j}}{2},\\ {\varepsilon }_{ij}=\sqrt{{\varepsilon }_{i}{\varepsilon }_{j}}\end{array}$$

**Table 1:** Lennard-Jones potential parameters used in this work and in previous studies. Cut-off radius *r*_*cut*_ for all cases in this study is 10.2 Å.

LJ parameter	This study	Ref.^36^	Ref.^37^
*σ*_*Ar*–*Ar*_, Å	3.400	3.405	3.406
*ε*_*Ar*–*Ar*_, meV	10.400	10.340	10.330
*σ*_*Ar*–*C*_, Å	3.400	3.380	3.380
*ε*_*Ar*–*C*_, meV	5.438	4.998	6.749

Carbon-carbon parameters ${\sigma }_{C-C}=3.4\,{\AA },{\varepsilon }_{C-C}=2.84\,meV$, that are used in combining rules, are taken from Lennard-Jones part of AIREBO potential^20^. The resulting parameters agree with the previously used ones (see Table 1).

A typical calculation consists of three stages: (i) construction of initial configuration, (ii) relaxation and (iii) statistical acquisition.

(i) The cubic box is used in the simulations. Firstly, two graphene layers are generated, they are stacked the same as in graphite: the bond length is 1.418 Å, the distance between layers is 3.35 Å. Then the upper graphene sheet is curved according to a previous experimental study^8^:
  2 $$h/H=1-{(r/R)}^{2}+\mathrm{0.25[(}r/R{)}^{2}-{(r/R)}^{4}]$$
  where *h* is the height at a particular point, and *r* is the radius at that point. The two graphene layers are consist of the same number of carbon atoms, hence the bonds in the upper layer are stretched. The corresponding average strain is of order ${(H/R)}^{2}\approx 1-\mathrm{2 \% }$. Finally, between the substrate and the 2D crystal, argon atoms in simple cubic lattice are inserted.
(ii) Calculations are performed using the software package, LAMMPS^23^. Periodic boundary conditions are applied in the in-plane (xy) directions. Non-periodic and fixed boundary conditions are applied in z direction, so that particles do not interact across the boundary and do not move from one side of the simulation box to the other. Two types of minimization were performed, MD and conjugate gradient descent, which yielded the same final results, except for the smallest nanobubble. In that case, the final results depend on the method of minimization, and we obtain two types of nanobubbles with the same number of argon atoms, regular and pancake. During the relaxation stage, the NPT ensemble is imposed on the substrate, and the force component perpendicular to the surface is set to zero. The target pressure for NPT ensemble is set to 0 bars, the target temperature for NPT ensemble is set to 300 K, which allows the relaxation of the stresses that occur in the substrate due to the initial artificial formation of the nanobubble. The 2D crystal and argon atoms are allowed to move in the NVT ensemble. All temperatures for NVT ensemble are set to 300 K. When the stresses in the substrate are removed, the substrate atoms are fixed, the 2D crystal and argon atoms continue to be set in the NVT ensemble. The MD time step for all calculations is set to 1 fs. The number of time steps to calculate one nanobubble varies from 5 to 20 million depending on the bubble size.
  It should be noted, although the initial shape of the nanobubble is set to the experimental one eq. 2, in general case the initial and final shapes of the nanobubble can differ significantly. The final shape is controlled by the number of inserted argon atoms. Thus the 2D crystal experiences strong fluctuations during the relaxation stage if the number of argon atoms is not equal to the equilibrium one (see Supplementary video 1). The final period of relaxation stage is shown at Supplementary video 2, structuring of argon atoms can be seen.
(iii) After the energy of the system stabilizes, we assume that equilibrium is achieved, and statistical information can be gathered. During the statistic acquisition stage, the NVT ensemble is imposed on the 2D crystal and argon atoms, the substrate atoms are fixed, and properties such as the shape of the nanobubble, structural properties of argon, and stresses in the 2D crystal are calculated. To evaluate the pressure inside the nanobubble, the volume is calculated using the Voronoi tessellation^24^. The per-atom stress tensor is evaluated according to ref.^25^. After averaging the per-atom stress tensor and obtaining the total stress tensor of the argon atoms, the pressure is calculated as a minus trace of the obtained stress tensor.

To analyze the obtained crystal structure of solid argon inside graphene nanobubbles, we use common neighbor analysis (CNA)^26,27^. The CNA uses a decomposition of the radial distribution function and provides direct interpretation of various features of the radial distribution function in terms of atomic structure. It can be used to identify atoms in particular environment, such as FCC, HCP, BCC or icosahedral.

Visualization is done by software package VMD^28^.

### Data Availability

The datasets generated during the current study are available from the corresponding author on reasonable request.

## Results and Discussion

**Figure 2:**
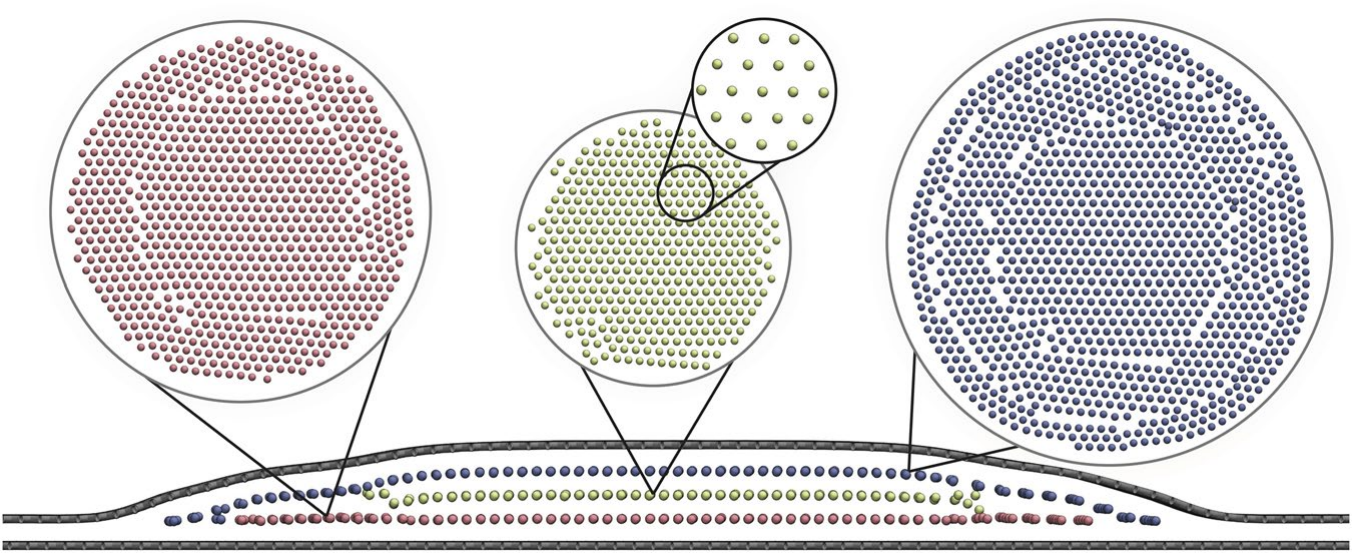
2D slice of the simulated graphene nanobubble with a radius of 7.3 nm and 3 layers of argon inside. Insets show the top view of each layer of argon and its hexagonal structure.

After relaxation, the argon inside all considered graphene nanobubbles with various radii is found to be in the solid state (see Fig. 2). For the largest bubble with a radius of 33.3 nm, CNA shows that 65% of the final crystal is face-centered cubic (FCC), 26% is hexagonal close-packed (HCP). Remaining 9% of undetermined atoms can be attributed to grain boundaries, defects and atoms that are in contact with 2D crystal or substrate. The argon atoms are layered perpendicular to the substrate. The spacing between argon layers for all simulated nanobubbles is approximately 3.05 ± 0.05 Å, and the in-layer distance between neighboring atoms is 3.7 ± 0.5 Å. These distances are directly calculated from atomic configurations during statistical acquisition stage of the modelling.

**Figure 3:**
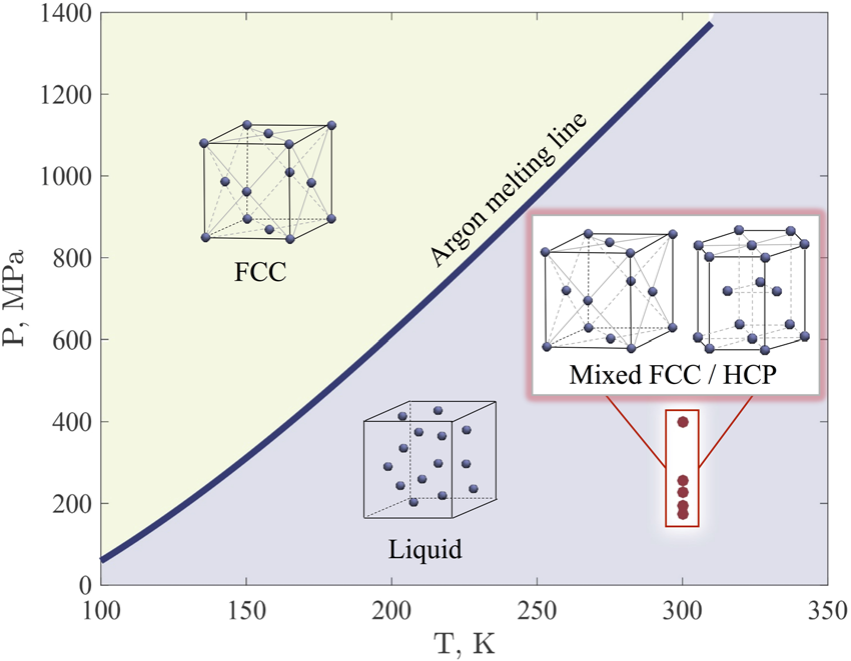
The melting line of bulk argon^29,30^ and the thermodynamic state of argon inside the graphene nanobubbles (red dots).

Table 2 shows the parameters of the simulated graphene nanobubbles. According to this table, all calculated pressures in the trapped substance are considerably lower than the melting pressure of bulk argon at 300 K, which is 1200 MPa^29,30^ (see Fig. 3). Therefore, in the case of argon atoms trapped inside nanobubbles, we observed additional stabilization that prevents the solid-to-liquid phase transition. We predict that further increasing the graphene nanobubble radius and the concomitant decrease in the argon pressure will eventually lead to the solid-to-liquid phase transition in the confined argon system. Figure 3 clearly shows that the melting line of argon in the nanobubble confinement (if one exist) should be located significantly below the melting line of bulk argon on P-T diagram.

**Table 2:** Parameters of the simulated graphene nanobubbles. R: radius; H: maximum height; N_Ar_: number of argon atoms inside the nanobubble; N_layers_: number of argon layers inside the nanobubble; *ρ*: argon density; P: argon pressure; L_x_, L_y_, L_z_: final lengths of the simulation box.

#	R, nm	H, nm	H/R	N_Ar_	N_layers_	*ρ*, g/cm^3^	P, MPa	L_x_, nm	L_y_, nm	L_z_, nm
1	7.8	0.64	0.083	2463	2	1.962	417	43.51	43.60	100
2	7.3	0.95	0.130	2463	3	1.911	398	43.55	43.60	100
3	13.8	1.57	0.114	12952	5	1.859	257	43.43	43.54	100
4	16.9	2.16	0.128	26153	7	1.849	229	78.77	79.17	100
5	24.0	2.77	0.116	73624	9	1.841	197	98.67	98.85	100
6	33.3	3.70	0.111	196331	12	1.837	172	98.55	98.83	100

The decrease in the pressure at which the melting takes place becomes even more noticeable if we consider the HCP phase of argon. The phase transition from the FCC argon to HCP argon occurs at pressures of several tens of GPa^31^ while calculated pressures inside graphen nanobubbles do not exceed 400 MPa. This can be explained by the fact that the specific free energy of HCP argon is very close to the specific free energy of FCC argon and metastable mixture of HCP/FCC could exist for very long time. HCP and FCC are close-packed structures and argon is inert atom without a designated direction of chemical bonding. Moreover, in solid argon in the graphene nanobubbles HCP structure is observed near surface of 2D crystal that could additionally lower its energy.

For the simulated nanobubbles, except for nanobubble 1, we observed a universal shape, i.e., a constant ratio between the maximum height and the radius. According to the elastic theory of membranes^8^ of round nanobubbles, this H/R ratio depends only on the material constants:

3 $$\frac{H}{R}={[\frac{\pi ({\gamma }_{GS}-{\gamma }_{GB}-{\gamma }_{SB})}{5cY}]}^{\frac{1}{4}}$$

where *H* is the maximum height of the nanobubble, *R* is the radius of the nanobubble, *γ*_*GS*_, *γ*_*GB*_, *γ*_*SB*_ are the adhesion energies between the graphene and substrate, the graphene and the substance inside the nanobubble, and the substrate and the substance, respectively, *Y* is Young’s modulus of graphene; and *c* is a dimensionless coefficient, which is 0.7 in the case of round nanobubbles.

To validate equation (3), Young’s modulus *Y* of graphene and the adhesion energies *γ*_*GS*_, *γ*_*GB*_, *γ*_*SB*_ are required. For consistency, all these parameters should be calculated from the potential used to model the nanobubble. Young’s modulus *Y* for the AIREBO potential was previously calculated^21^ to be 21.12*eV*/Å^2^ (1.01*TPa*), which agrees well with *ab initio* calculations and experiments.

The adhesion energy between the graphene and the substrate is calculated as the work required to separate graphene from the substrate. For the AIREBO potential, we obtain a value of ${\gamma }_{GS}=0.017eV/{{\rm{\AA }}}^{2}$, which is the typical adhesion energy of graphene membranes^32^. In our case, *γ*_*GB*_ = *γ*_*SB*_ because the 2D crystal and substrate consist of the same material. This adhesion energy is also calculated in this work. Three layers of graphene in contact with argon atoms are considered. The potential energy *U*_*composite*_ of this system is also calculated. Then, only argon atoms are considered at the same pressure and the same temperature of *T* = 300 *K*, and their potential energy *U*_*argon*_ is evaluated. Finally, the potential energy of the three layers of graphene, *U*_*graphene*_, is computed. Adhesion energies *γ*_*GB*_ and *γ*_*SB*_ are evaluated as (*U*_*composite*_ − *U*_*argon*_ − *U*_*graphene*_)/*S*_*area*_. For two different argon pressures, 86 *MPa* and 291 *MPa*, we calculate *γ*_*GB*_ = *γ*_*SB*_ to be 0.0049 *eV*/Å^2^ and 0.0052 *eV*/Å^2^, respectively. Because these values are very similar, for estimation, we take the average of these two values, ${\gamma }_{GB}={\gamma }_{SB}=0.0051\,eV/{{\rm{\AA }}}^{2}$. Thus, for values of $Y=22.12\,eV/{{\rm{\AA }}}^{2}$, ${\gamma }_{GS}=0.017\,eV/{{\rm{\AA }}}^{2}$, and ${\gamma }_{GB}={\gamma }_{SB}=0.0051\,eV/{{\rm{\AA }}}^{2}$ that are obtained purely by MD, the elastic theory of membranes according to equation (3) gives H/R = 0.128.

The calculated values of the H/R ratio for nanobubbles 2–6 (see Table 2) are within 10% of 0.128, which is the value obtained using the elastic theory of membranes. This result confirms the applicability of this theory to interpreting atomic-force microscope measurements.

**Figure 4:**
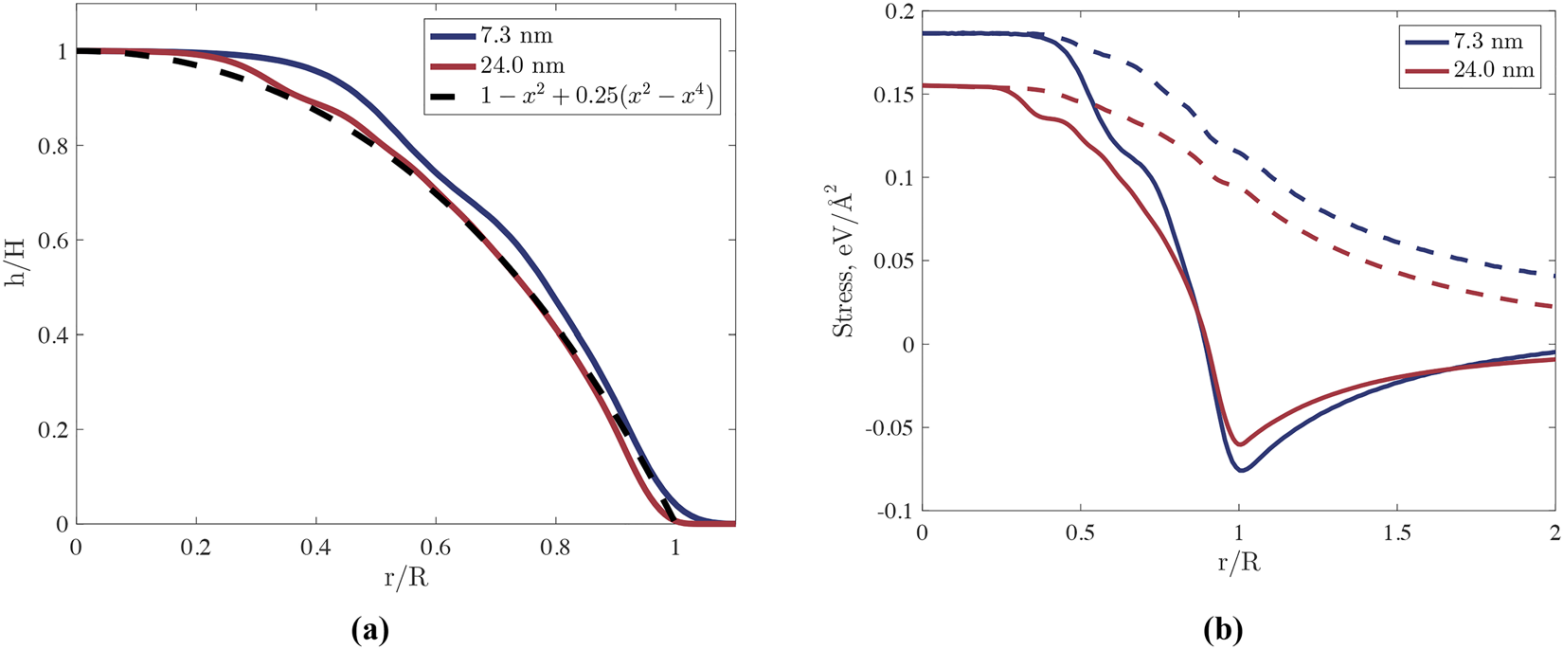
(**a**) Dashed black line: shape of the graphene nanobubble in dimensionless coordinates taken from^8^; blue line: shape of the graphene nanobubble with R = 7.3 nm obtained from MD; red line: shape of the graphene nanobubble with R = 24.0 nm obtained from MD. (**b**) Distribution of stress *σ*_*rr*_ (solid lines) and *σ*_*θθ*_ (dashed lines) along the radial direction. Blue lines: graphene nanobubble with R = 7.3 nm; red lines: graphene nanobubble with R = 24.0 nm.

Figure (4a) shows the shape of the obtained graphene nanobubbles in dimensionless coordinates compared with the dimensionless shape given by the elastic theory of membranes. The results obtained using MD calculations agree with those obtained from the elastic theory of membranes. The slight difference is explained by the fact that the elastic theory of membranes has not considered that a solid structure inside the bubble can lead to a stepped morphology. In addition, in the elastic theory of membranes, bending rigidity is neglected. Therefore, the analytic curve is “stuck” at zero at *r* = *R*, whereas that calculated from the MD curve smoothly approaches zero. Additionally, the distribution of stress along the radial direction is calculated, and the radial and angular components of the stress are presented in Fig. (4b). The overall form of the curves coincides with the dimensionless form obtained via the elastic theory of membranes.

AFM studies^8^ of graphene nanobubbles do not give information about structure of a trapped material. Combined scanning tunneling microscopy (STM), low energy electron microscopy (LEEM) and synchrotron-based photoemission electron microscopy (XPEEM) make possible to investigate both the shape of the nanobubbles and the structure of trapped material. Experimental research of graphene nanobubbles filled with argon on Ir^14^ provides direct evidence of argon cluster formation at and above room temperature which supports the existence of solid argon inside graphene nanobubble. The direct comparison between results of this work and experimental study^14^ can not be done due to fundamental difference in types of substrate. In our cause the substrate is graphene and interaction between 2D crystal and substrate governed by Van der Waals forces. When substrate is metallic as in study^14^ the interaction between the 2D crystal and the substrate has a complex nature related to the redistribution of the charge density between atoms^10^.

Argon is solid at room temperature at a pressure which is significantly lower than that shown by the P-T diagram (see Fig. 3) and than expected for the condensation of compressed argon. This fact could be interpret from the point of view of well-known physical phenomenon of multilayer adsorption. If argon exist in gaseous or liquid state and is in contact with graphene sheet, structured layers of argon arise at the interface. Monte Carlo simulation of argon adsorption on graphite^33^ shows that at pressure 100 MPa and temperature 260 K approximately three argon layers are constructed on the substrate. These pressure and temperature are close to the parameters in largest nanobubbles that is simulated (see Table 2). In case of the graphene nanobubbles there are two surfaces that can adsorb argon atoms and hence at least 6 layer have to arise at this conditions. Therefore it is not so unexpected that argon solidify inside the graphene nanobubbles at lower pressures in comparison with bulk argon.

**Figure 5:**
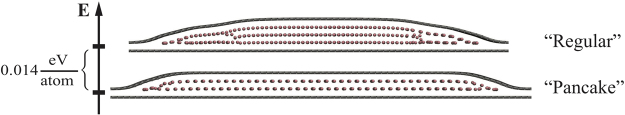
2D slice of “regular” and “pancake” graphene nanobubbles on the energy scale.

Nanobubble 1 in Table 2 significantly differs from the others in terms of its small ratio of H/R = 0.083. Such a small ratio indicates that this type of nanobubble is flatter than others. We will refer to this exotically shaped graphene nanobubble as a “pancake” graphene nanobubble. A pancake nanobubble was obtained during the initial configuration setup (see Methods for more details) when a conjugate gradient minimization of energy was used instead of MD minimization. For a higher number of atoms inside the nanobubbles, both minimization algorithms lead to the same final “regular” form. The number of argon atoms in graphene nanobubbles 1 and 2 (see Table 2) is the same, but they exhibit different geometries, e.g., different numbers of layers of solid argon. Additionally, the pancake nanobubble has a lower energy than the regular nanobubble with the same number of argon atoms inside (see Fig. 5). To the best of our knowledge, this is the first report of the possible existence of a pancake nanobubble in heterostructures. Pancake nanobubbles have been observed in a completely different system at the interface between highly oriented pyrolytic graphite and water^34,35^. We assume that this suggests that these two systems are highly analogous. The emergence of the pancake shape is a manifestation of the complicated interplay between surface interactions and the inner state of trapped substances.

## Conclusions

Argon trapped in small graphene nanobubbles with a radius of less than 33 nm does exist in a solid close-packed phase with a mixture of FCC and HCP lattices. The pressure of argon in these nanobubbles is considerably lower than the pressure required for the ordinary crystallization to occur in a bulk system. Therefore, these objects present good examples of physical systems in which the phase state is highly affected by interface interactions. Compared with nanotubes, which can only accommodate a few dozen atoms inside, the heterostructured nanobubbles can accumulate millions of atoms and molecules, and therefore can be treated as true thermodynamic systems. In this work, we observed a layered structure of argon atoms inside graphene nanobubbles. The shape of the observed nanobubbles, except for the smallest one, obeyed a universal shape law, i.e., a constant ratio of height to radius, independent on the nanobubble radius. This is consistent with the elastic theory of membranes^8^, which was previously used to describe the morphology of graphene nanobubbles. The dimensionless shape and the stress distribution calculated from MD are in good agreement with the elastic theory of membranes. We also found very small nanobubbles with an exotic pancake shape that has not been reported so far in the case of heterostructured nanobubbles. However, this shape has been observed for nanobubbles at the interface between water and highly oriented pyrolytic graphite.

We expect that a subsequent increase in the nanobubble radius will lead to a pressure drop inside nanobubble and will eventually lead to the solid-liquid phase transition in the confined argon system. This research clearly demonstrates the possibility that nanobubbles in heterostructures can be used to investigate the fundamentally interesting phenomenon of phase transition in confined systems.

## Electronic supplementary material

Side view of considered nanobubbles

Video 1

Video 2
