# Supplementary material for: Atomistic study of the solid state inside graphene nanobubbles
Source: Sci Rep. 2017 Dec 20;7:17906. doi: 10.1038/s41598-017-18226-9 (PMC5738358; doi:10.1038/s41598-017-18226-9)
Supplement: Supplementary file 1 — Side view of considered nanobubbles [file 41598_2017_18226_MOESM1_ESM.pdf]

Supplementary information for manuscript “Atomistic study of the solid state inside graphene nanobubbles”

Evgeny Iakovlev, Petr Zhilyaev, and Iskander Akhatov

	R, nm	H, nm	N_{Ar}
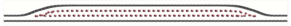	7.8	0.64	2463
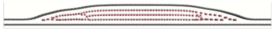	7.3	0.95	2463
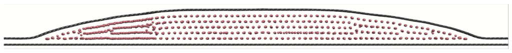	13.8	1.57	12952
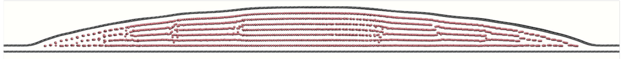	16.9	2.16	26153
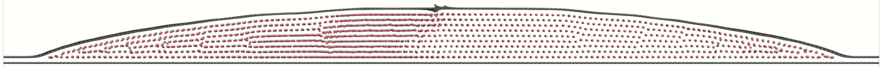	24.0	2.77	73624
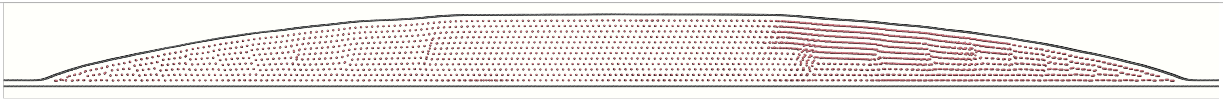	33.3	3.70	196331

Table 1. Side view of considered graphene nanobubbles filled with argon at T=300 K (in scale).
